# Combined Effect of Secondhand Smoking and Alcohol Drinking on Risk of Persistent Human Papillomavirus Infection

**DOI:** 10.1155/2019/5829676

**Published:** 2019-03-21

**Authors:** Sang-Soo Seo, Hea Young Oh, Mi Kyung Kim, Dong Ock Lee, Youn Kyung Chung, Joo-Young Kim, Chan Wha Lee

**Affiliations:** ^1^Center for Uterine Cancer, National Cancer Center, 323, Ilsan-ro, Ilsandong-gu, Goyang-si 10408, Republic of Korea; ^2^Division of Cancer Epidemiology and Prevention, National Cancer Center, 323, Ilsan-ro, Ilsandong-gu, Goyang-si 10408, Republic of Korea; ^3^Center for Cancer Prevention and Detection, Hospital, National Cancer Center, 323, Ilsan-ro, Ilsandong-gu, Goyang-si 10408, Republic of Korea

## Abstract

Tobacco smoking is established as a cofactor of human papillomavirus (HPV) for cervical cancer risk. However, the role of secondhand smoking in cervical carcinogenesis is controversial. We aimed to assess the association between secondhand smoking and high risk- (HR-) HPV persistence, a pivotal event in development of cervical cancer. In total, 9,846 women who underwent health-screening examinations from 2002 to 2011 at the National Cancer Center, Korea, were included. Secondhand smoking was defined as being exposed to secondhand smoke at home or in the workplace. Multivariate logistic regression analysis was used to estimate the odds ratios (OR) and 95% confidence intervals (CIs) for risks of HR-HPV infection at baseline (N, 9,846, negative vs. positive), 1-year persistence (n, 1,237, 1-year negative vs. 1-year persistence), and 2-year persistence (n, 481, 2-year negative vs. 2-year persistence). Active smoking, secondhand smoking, and secondhand smoking in nonactive smokers had no association with these risks. Among alcohol drinkers, secondhand smoking in nonactive smokers had higher risks of HR-HPV infection at baseline (OR = 1.25, 95% CI = 1.05–1.48,* p* for multiplicative interaction = 0.003), 1-year persistence (1.75, 1.14–2.68, 0.004), and 2-year persistence (2.96, 1.42–6.15, 0.006), when compared to HR-HPV negative, 1-year negative, and 2-year negative categories, respectively. However, among nonalcohol drinkers, there was no association between smoking or secondhand smoking status and these risks. These findings suggest that women exposed to secondhand smoking at home or in the workplace might be at high risk of HR-HPV persistence when it is combined with alcohol drinking, even though neither active smoking nor secondhand smoking independently affects the risk.

## 1. Introduction

Several studies, including a large meta-analysis and several cohort studies, have shown an association between cigarette smoking and cervical cancer or its precursor lesions [1–3]. Cigarette smoking is established as a cofactor of human papillomavirus (HPV) for cervical cancer [1]. However, the role of secondhand smoking (SHS) therein is controversial. One study, based on a pooled analysis of couples from the International Agency for Research on Cancer (IARC) multicenter case-control studies, suggested that SHS could not be an independent risk factor for invasive cervical cancer in the absence of active smoking [2]. In contrast, other studies have shown a potential impact of SHS on the risk of cervical neoplasia [2–9]. With this background, no study has thoroughly examined the association between exposure to SHS and persistent infection of HPV, a critical initial event in progression to cervical neoplasia [10].

In the Republic of Korea, cigarette smoking among women is rare (7% in 2009), but the proportion of men aged ≥ 15 years who smoke is the second highest among the Organization for Economic Cooperation and Development (OECD) countries [11]. Similar tobacco-use patterns are found in other Asian countries, where studies have shown the potential effect of SHS on the risk of cervical neoplasia [5, 7, 9]. Although there are no reports on the association between SHS and development of cervical cancer in Korean women, several studies have shown that exposure to SHS was positively associated with incidence of lung cancer in nonsmoking women whose husbands smoked [12] and with osteoporosis in postmenopausal never-smoking women [13].

Although most studies have investigated cervical cancer precursors, we have focused on the initiation of cervical carcinogenesis (i.e., persistent HPV infection) in order to aid efforts at primary prevention of cervical cancer. In the present study, we aimed to assess the associations between smoking status (e.g., active smoking, SHS, and SHS in nonsmokers) and the risks of HR-HPV infection at baseline, 1-year HR-HPV persistence, and 2-year HR-HPV persistence as well as the same associations among alcohol drinkers.

## 2. Materials and Methods

### 2.1. Study Design and Population

**Figure 1:**
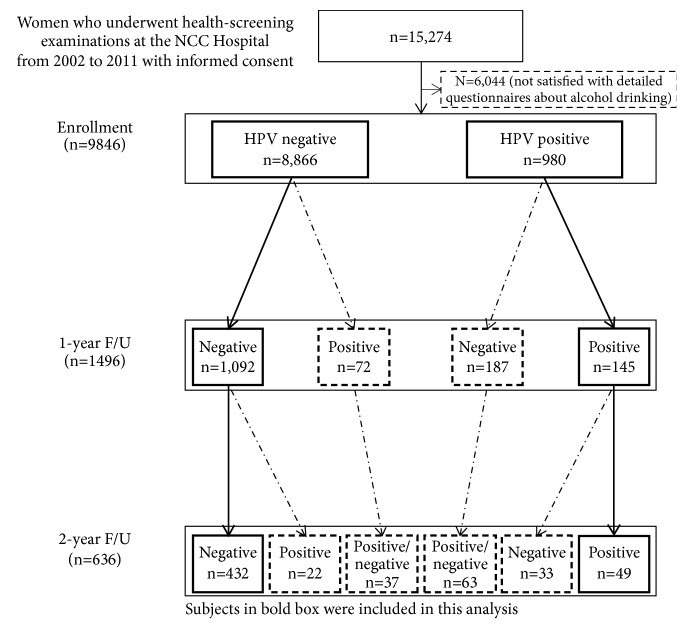
Subjects in bold boxes included in present analysis. “Negative” or “Positive” indicates the result of detection for 13 DNA types of high risk-human papillomavirus (HR-HPV) (16, 18, 31, 33, 35, 39, 45, 51, 52, 56, 58, 59, and 68) using Hybrid Capture II.

This study was a part of the Korean Prospective Study for the Transition of Human Papillomavirus into Cervical Carcinoma [14, 15]. Among women who provided signed informed consent and underwent health-screening examinations at the National Cancer Center from 2002 to 2011, a total of 9,846 who had results of HR-HPV DNA detection and complete questionnaire responses regarding smoking behaviors (e.g., active cigarette smoking and SHS) were included in this study. We collected the results of HR-HPV DNA detection and cytology findings (Papanicolaou smear test) and administered comprehensive lifestyle questionnaires. The questionnaire collected data on age, height, weight, smoking behaviors, alcohol behaviors, parity, marital status, education level, menopausal status, and oral contraceptive use. Of the 9,846 women, all were included in the analysis for HR-HPV infection at baseline, 1,496 were available for 1-year follow-up after excluding 29 women who had HSIL or more severe cytological results at baseline, and 636 were available for 2-year follow-up (Figure 1). Among the 9,846 women at baseline, 980 were HR-HPV positive and 8,866 were HR-HPV negative. In the follow-up study, 145 and 49 women were included in the 1- and 2-year HPV persistence categories and 1,092 and 432 women were included in the 1- and 2-year HPV negative categories, respectively.

### 2.2. Ethics, Consent, and Permissions

This study received approval from the Institutional Review Board of the National Cancer Center, Korea (NCCNCS-11-433), and all of the study participants provided informed consent.

### 2.3. Questionnaires Related to Smoking Behaviors

Information on smoking and secondhand smoking behaviors was collected using a standardized questionnaire (*supplement*). All of the participants were asked if they had ever smoked a cigarette.* Nonsmokers were defined as having smoked <100 cigarettes in their lifetimes (n = 9,021; 5739 with SHS, 3282 without). Smokers were defined as having smoked ≥100 cigarettes in their lifetimes (n = 825)*. Regarding SHS, all of the participants were asked, “Do you live with anyone in your household who regularly smokes cigarettes or other tobacco products (other than yourself)?” and “Do you work with anyone in your workplace who regularly smokes cigarettes or other tobacco products (other than yourself)?” (1) Exposure to SHS at home = No/Yes, how many family persons who smoked regularly, time of exposure to SHS; (2) exposure to SHS in the workplace = No/Yes, time of exposure to SHS. We did not account for exposure to SHS in public places. Secondhand smoking was defined as being exposed to SHS in either the home or the workplace,* regardless of active smoking status*, while non-SHS was defined as not being exposed to SHS at home or in the workplace.* In addition, SHS in nonsmokers was defined as having never smoked in life but having been exposed to SHS*.

### 2.4. HPV DNA Detection and Pap Smear

HR-HPV DNA was detected using the commercially available Hybrid Capture II system (HC II; Digene Co., Silver Spring, MD, USA). A chemiluminescent HPV DNA test was used to measure relative light units (RLU) with a probe designed for 13 types of HR-HPV. The test results were read as positive at concentrations of 1 pg/ml or greater than the RLU/cutoff ratio (RLU of specimen/mean RLU of two positive controls). The cytological grades for Pap smear reports were based on the Bethesda classification system [16].

### 2.5. Statistical Analysis

Descriptive statistics were used to summarize the overall distribution of characteristics by study group. The chi-square test and* t*-test were used to assess the differences in the distributions of categorical and continuous variables between groups, respectively. Multivariate logistic regression models were used to estimate odds ratios (ORs) and corresponding 95% confidence intervals (CIs). A multivariate logistic regression analysis was run to assess the associations between smoking status and risk of HR-HPV infection at baseline, 1-year persistence, and 2-year persistence after adjustment for confounding factors. We checked for confounding by calculating the Mantel-Haenszel summary measure for the effect of alcohol drinking status. After separating data for strata defined for alcohol drinkers and nonalcohol drinkers, a multivariate logistic regression analysis was again performed to assess the association between smoking status and risk. All of the analyses were adjusted for age and BMI continuously and for marital status, parity, menopausal status, oral contraceptive use, education level, and alcohol drinking status as well. Risk estimates were calculated for nonactive smoking, non-SHS smoking, and non-SHS smoking in nonactive smokers as reference categories. All of the analyses were performed with SAS 9.3 (SAS Institute, Cary, NC, USA) and the episensr packages in R language (version 3.3.2) [17].

## 3. Results

### 3.1. General Characteristics of Study Subjects

Of the 9,846 women, 980 (9.9%) were infected by HR-HPV at enrollment (Supplementary Table 1). The mean age of the subjects was 48, and the mean BMI was 23.0 kg/m^2^. Most of the subjects were married (97%) and had two or more children (87.3%). Approximately 60% of the subjects were postmenopausal women and 20% had used oral contraceptives (currently or in the past). Approximately one-half of the subjects had consumed alcoholic beverages (currently or in the past), and 8 and 37% were active cigarette smokers and secondhand smokers, respectively. At enrollment, the majority of the subjects (97%) had normal cytological results, but 186 (2.0%) had atypical squamous cells of undetermined significance (ASCUS), 58 (0.6%) had low-grade squamous intraepithelial lesions (LSIL), and 29 (0.3%) had high-grade squamous intraepithelial lesions (HSIL) or more severe abnormalities. There were wide variations in the distributions of age, BMI, menopausal status, education level, alcohol drinking status, and active smoking status. These confounding factors were adjusted in a subsequent multivariate logistic regression analysis.

### 3.2. Association between Smoking Status and HR-HPV Infection or Its Persistence

We assessed the associations between active smoking, SHS, and SHS in nonsmokers who had never smoked in their lives and the risk of HR-HPV infection and its persistence. There were no significant associations between smoking status (active smoking, SHS, or SHS in nonactive smokers) and the risks of HR-HPV infection at enrollment, 1-year HR-HPV persistence, and 2-year HR-HPV persistence (Table 1). Only alcohol drinking showed a high risk of 2-year HR-HPV persistence (multivariate OR= 3.08, 95% CI=1.22–7.77).

### 3.3. Combined Effect of SHS and Alcohol Drinking on HR-HPV Persistence Risk

Previously, alcohol drinking behaviors and their combination with high HR-HPV viral load were associated with risk of HR-HPV persistence [14, 15]. The Mantel-Haenszel statistics showed a significant alcohol drinking status difference in the OR of SHS for 1-year HR-HPV persistence (*p* = 0.038, homogeneity of the OR). The subjects were stratified by alcohol drinking status (nondrinkers/current or past drinkers), and the associations between smoking status and the risks of HR-HPV infection and its persistence were assessed among alcohol drinkers and nondrinkers, separately (Table 2). Among alcohol drinkers, active smoking had no association with these risks, but SHS had higher risks of HR-HPV infection at baseline (OR = 1.25, 95% CI = 1.06–1.47,* p* for multiplicative interaction = 0.005), 1-year HR-HPV persistence (1.64, 1.09–2.48, 0.012), and 2-year HR-HPV persistence (2.54, 1.25–5.17, 0.029). Secondhand smoking in nonactive smokers also had higher risks of HR-HPV infection at baseline (OR = 1.25, 95% CI = 1.05–1.48,* p* for multiplicative interaction = 0.003), 1-year HR-HPV persistence (1.75, 1.14–2.68, 0.004), and 2-year HR-HPV persistence (2.96, 1.42–6.15, 0.006). Among alcohol nondrinkers, active smoking had no association with these risks, but SHS had lower risks of HR-HPV infection at baseline (OR = 0.70, 95% CI = 0.56–0.87) and 1-year HR-HPV persistence (0.40, 0.21–0.77). SHS in nonactive smokers also had lower risks of HR-HPV infection at baseline (0.78, 0.64–0.95) and 1-year HR-HPV persistence (0.43, 0.23–0.77).

## 4. Discussion

Our findings showed that the combination of exposure to SHS and alcohol drinking was associated with higher risks of HR-HPV infection and its persistence in women who had never smoked in their lives but had been exposed to SHS.* Ours is the first report of an association between SHS and HR-HPV persistence and the combined effect of SHS and alcohol drinking on that risk*. Whereas most of the previous studies focused on cervical cancer precursors [10], our present study targeted the step before the onset of neoplasia in order to facilitate primary prevention of cervical cancer. Furthermore, ours is a large-scale study that utilized health-screening examinations and made not only cross-sectional but also prospective observations; in short, our findings might be more credible, especially due to the present study's thorough assessment of the causal relationships between risk factors and outcome status.

Tobacco smoking has been established as an important risk factor in cervical carcinogenesis [1, 18–21] and oral HPV infection [22, 23]. However, even though there are several reports on the effects of SHS [2–9, 24], the association between SHS and cervical carcinogenesis has never been clearly evaluated. Persistent infection with HR-HPV genotypes (13 oncogenic) is the first in the following series of steps leading to cervical cancer: HPV infection of metaplastic epithelium, viral persistence, progression to a cervical precancerous lesion, and invasion [10]. Exposure to SHS has been reported to be an important independent risk factor for cervical epithelial abnormalities [3, 6, 8] and cervical cancer [25] in the United States. Similarly, SHS has been associated with the risk of cervical neoplasia in several Asian countries such as Taiwan, Singapore, and Thailand [4, 5, 7, 9]. A recent meta-analysis provided evidence that passive smoking is associated with an increased risk of cervical cancer [24]. However, a pooled study including couples in seven case-control studies reported that SHS was not an independent risk factor of invasive cervical cancer in the absence of active smoking. A significantly increased risk of invasive cervical cancer with SHS was found among all couples in the pooled study, but not among nonsmoking women married to ever-smoking men [2]. Tobacco smoking among Korean women is rare (female smokers aged ≥19 years: 6.8% in 2011), and the rates of women exposed to SHS at home (16.7% in 2011) and in the workplace (37.2% in 2011) were higher than that of active woman smokers [26]. Although there is a lack of evidence of any association between cervical neoplasia and SHS smoking in Korean women, several reports have demonstrated a potential effect of SHS on the onset of lung cancer, femoral neck osteoporosis, and type 2 diabetes [12, 13, 27].

As these findings suggest, exposure to SHS might be related to the development of cervical neoplasia in Korea women. A previous study showed that alcohol drinking is associated with a higher risk of HR-HPV persistence in Korean women [14]. It is certainly true that alcohol drinking status might be a more critical cofactor than SHS. However, in the present study, although exposure to SHS did not have an independent effect on the risk, alcohol drinkers who had been exposed to SHS showed higher HR-HPV persistence than did alcohol drinkers who had not been so exposed. Evidence showing that the combination of HPV and alcohol drinking might contribute to progression of cervical carcinogenesis has been reported in previous studies in which alcohol drinkers with a high HPV viral load (≥ 100 RLU/PC) had a higher risk of HR-HPV persistence [15] and cervical intraepithelial neoplasia 1 grade (OR: 19.1) [28]. Hassan et al. also demonstrated that regular cigarette smoking was not associated with hepatocellular carcinoma in women but that cigarette smoking did act synergistically with heavy alcohol consumption in women (OR: 13.7), thereby increasing the risk of hepatocellular carcinoma [29].

There are plausible mechanisms explaining the impact of SHS on risk of HPV persistence. Tobacco includes carcinogens that could have a direct transformational effect on the cervix, leading to immune suppression and HPV persistence [2, 30]. Actually, nicotine and other components of tobacco smoke have been found in the mucus of the cervical epithelium of nonsmokers [3]. Direct cervical contact with mutagenic semen of active smoking partners could also be a plausible source of exposure in secondhand smokers [31, 32]. Alcohol itself is involved in cancer development through cytochrome p450 2E1, leading to the generation of oxidative stress [33]. Various antioxidant enzymes and detoxifying pathways are consistently associated with HPV-transformed cells [34, 35]. Thus, both nicotine and alcohol are established as critical carcinogens [36] as well as behavioral factors that weaken the immune system [37]. Therefore too, it is likely that the combination of nicotine and alcohol has a synergistic effect on viral persistence.

Our study has several limitations. First, although sexual behaviors are critical confounding factors of HPV infection [1], bias due to this unmeasured confounder was not fully analyzed, due to the absence of this topic in the health-screening questionnaire utilized. Sensitivity analyses of potential bias [38] due to unmeasured confounders suggested that observed SHS-HPV persistence association is probably not entirely due to confounding by sexual behaviors. However, those analyses did not fully address the issue of confounding by sexual behaviors; thus, we need to confirm our observed SHS-HPV persistence association in other settings with full measurement of potential confounders. In any case, we could not exclude the possibility that our observed associations with SNS among alcohol drinkers in this study were confounded by sexual behavior, due particularly to the plausible relationships of alcohol consumption and combined smoking/alcohol use with acquisition of HPV through increased sexual activity among alcohol drinkers [39, 40]. The second limitation of this study is the fact that it did not use data on the specific HPV genotype. However, multiple-type HR-HPV infection in Korean women is very low: single- and multiple-type infections among HR-HPV-infected women were 8.3 and 0.5%, respectively [41]. Furthermore, pooled detection of HR-HPV genotypes has been reported to be more sensitive for CIN 3 than for genotype-specific persistence and can minimize false negative results [42, 43]. Third, this study had a relatively large loss-to-follow-up ratio between the baseline and follow-up visits. This could have had an impact on the results through the introduction of potential selection biases. Fourth and finally, whereas we administered detailed questionnaires to ascertain smoking status and SHS-related variables such as first age of exposure to smoking behaviors, durations of exposure, and the exposure environment, the numbers of women who completed these questionnaires were very small. However, questionnaires with an insufficient number of responses (duration of exposure and first age of exposure) were excluded from the analysis. Thus, active smoking and SHS might have been underestimated or overestimated in this study. This limitation will be addressed by further studies using our ongoing cohort.

## 5. Conclusions

In conclusion, alcohol drinkers exposed to SHS at home or in the workplace had a higher risk of persistent HR-HPV infection than did alcohol drinkers not so exposed. This combined effect was observed also in women who had never smoked in their lives. Reducing both exposure to SHS and consumption of alcoholic beverages could be a prudent approach to the prevention of cervical cancer.

## Figures and Tables

**Table 1:** Odds ratios of active smoking and secondhand smoking for risk of HR-HPV infection and HR-HPV persistence.

	*HR-HPV infection at enrollment* (n = 9,846)			*1-year HR-HPV persistence* (n = 1,237)			*2-year HR-HPV persistence* (n = 481)		
	Negative^a^	Positive^a^	Multivariate OR	Negative^a^	Persistence^a^	Multivariate OR	Negative^a^	Persistence^a^	Multivariate OR
Characteristics	(n = 8,866)	(n = 980)	(95% CI)^b^	(n = 1,132)	(n = 145)	(95% CI)^b^	(n = 432)	(n = 49)	(95% CI)^b^
Active smoking									
Nonsmoking	8,145 (90.3)	876 (9.7)	1 (ref.)	1,002 (88.5)	130 (11.5)	1 (ref.)	388 (89.4)	46 (10.6)	1 (ref.)
Smoking	721 (87.4)	104 (12.6)	1.18 (0.94–1.48)	90 (85.7)	15 (14.3)	0.98 (0.53–1.80)	44 (93.6)	3 (6.4)	0.38 (0.11–1.37)
Secondhand smoking (SHS)									
Non-SHS	5,628 (90.4)	601 (9.6)	1 (ref.)	672 (88.4)	88 (11.6)	1 (ref.)	251 (89.6)	29 (10.4)	1 (ref.)
SHS^c^	3,238 (89.5)	379 (10.5)	1.01 (0.88–1.17)	420 (88.1)	57 (11.9)	0.86 (0.59–1.23)	181 (90.1)	20 (9.9)	0.95 (0.51–1.78)
SHS in nonsmokers ^d^									
Non-SHS in nonsmokers	5,199 (90.6)	540 (9.4)	1 (ref.)	619 (88.7)	79 (11.3)	1 (ref.)	230 (89.5)	27 (10.5)	1 (ref.)
SHS in nonsmokers	2,946 (89.8)	336 (10.2)	1.00 (0.86–1.16)	383 (88.3)	51 (11.7)	0.89 (0.62–1.29)	158 (89.3)	19 (10.7)	1.14 (0.61–2.13)
Alcohol drinking									
Nondrinker	4242 (91.2)	410 (8.8)	1 (ref.)	580 (90.8)	59 (9.2)	1 (ref.)	231 (91.7)	21 (8.3)	1 (ref.)
Drinker	4056 (88.7)	519 (11.3)	1.19 (0.97–1.46)	447 (84.8)	80 (15.2)	1.44 (0.87–2.39)	177 (86.3)	28 (13.7)	3.08 (1.22–7.77)

^a^ “Negative” or “Positive” indicates the result of detection for 13 DNA types of high risk-human papillomavirus (HR-HPV) (16, 18, 31, 33, 35, 39, 45, 51, 52, 56, 58, 59, and 68) using Hybrid Capture II.

^b^ All variables were adjusted for age (as continuous) and BMI (as continuous) as well as for marital status, oral contraceptive use, menopausal status, education level, number of children, active smoking status (for alcohol drinking), passive smoking (for alcohol drinking), and alcohol drinking status (for smoking).

^c^ Non-SHS indicates persons who are not exposed to secondhand smoking at home or in the workplace, regardless of active smoking status.

^d^ Secondhand smoking (SHS) in nonsmokers was defined as having never smoked in life but having been exposed to SHS.

**Table 2:** Odds ratios for risk of HPV persistence of active smokers and secondhand smokers according to alcohol drinking status.

	*HR-HPV infection at enrollment* ^a^ (n = 4,578)			*1-year HR-HPV persistence* ^b^ (n = 527)			*2-year HR-HPV persistence* ^b^ (n = 205)		
*Alcohol drinkers*	Negative	Positive	Multivariate OR	Negative	Persistence	Multivariate OR	Negative	Persistence	Multivariate OR
	(n = 4,059)	(n = 519)	(95% CI)^e^	(n = 447)	(n = 80)	(95% CI) ^e^	(n = 177)	(n = 28)	(95% CI) ^e^

Active tobacco smoking									
Nonsmoking	3527 (88.9)	440 (11.1)	1 (ref.)	386 (84.8)	69 (15.2)	1 (ref.)	144 (84.7)	26 (15.3)	1 (ref.)
Smoking	532 (87.1)	79 (12.9)	1.14 (0.88–1.49) ^2)^	61 (84.7)	11 (15.3)	0.92 (0.45–1.92)	33 (94.3)	2 (5.7)	0.30 (0.07–1.43)
*p* for interaction^f^			0.072 ^3)^			0.586			0.257
Secondhand smoking (SHS)									
Non-SHS	2402 (89.4)	285 (10.6)	1 (ref.)	250 (86.5)	39 (13.5)	1 (ref.)	98 (88.3)	13 (11.7)	1 (ref.)
SHS ^c^	1657 (87.6)	234 (12.4)	1.25 (1.06–1.47)	197 (82.8)	41 (17.2)	1.64 (1.09–2.48)	79 (84.0)	15 (16.0)	2.54 (1.25–5.17)
*p* for interaction^f^			0.005			0.012			0.029
SHS in nonactive smokers									
Non-SHS	2092 (89.7)	240 (10.3)	1 (ref.)	218 (86.9)	33 (13.1)	1 (ref.)	82 (87.2)	12 (12.8)	1 (ref.)
SHS ^d^	1435 (87.8)	200 (12.2)	1.25 (1.05–1.48)	168 (82.4)	36 (17.6)	1.75 (1.14–2.68)	62 (81.6)	14 (18.4)	2.96 (1.42–6.15)
*p* for interaction^f^			0.003			0.004			0.006

	*HR-HPV infection at enrollment* ^a^ (n =4,652 )			*1-year HR-HPV persistence* ^b^ (n =639)			*2-year HR-HPV persistence* ^b^ (n =252)		
*Alcohol nondrinkers*	Negative	Positive	Multivariate OR	Negative	Persistence	Multivariate OR	Negative	Persistence	Multivariate OR
	(n = 4,242)	(n = 410)	(95% CI)	(n = 580)	(n = 59)	(95% CI)	(n = 231)	(n = 21)	(95% CI)

Active tobacco smoking									
Nonsmoking	4076 (91.3)	387 (8.7)	1 (ref.)	555 (91.0)	55 (9.0)	1 (ref.)	222 (91.7)	20 (8.3)	1 (ref.)
Smoking	166 (87.8)	23 (12.2)	1.26 (0.81–1.96)	25 (86.2)	4 (13.8)	1.20 (0.39–3.65)	9 (90.0)	1 (10.0)	0.89 (0.11–7.48)
Secondhand smoking (SHS)									
Non-SHS	2806 (91.0)	279 (9.0)	1 (ref.)	379 (89.4)	45 (10.6)	1 (ref.)	137 (89.5)	16 (10.5)	1 (ref.)
SHS ^c^	1436 (91.6)	131 (8.4)	0.70 (0.56–0.87)	201 (93.5)	14 (6.5)	0.40 (0.21–0.77)	94 (95.0)	5 (5.0)	0.52 (0.20–1.39)
SHS in nonactive smokers									
Non-SHS	2701 (91.1)	265 (8.9)	1 (ref.)	361 (89.6)	42 (10.4)	1 (ref.)	134 (89.9)	15 (10.1)	1 (ref.)
SHS ^d^	1375 (91.8)	122 (8.2)	0.78 (0.64–0.95)	194 (93.7)	13 (6.3)	0.43 (0.23–0.77)	88 (94.6)	5 (5.4)	0.49 (0.19–1.29)

^a^ Enrollment HR-HPV infection indicates the HR-HPV infection at the time of enrollment. “Negative” or “Positive” indicates the result of detection for 13 DNA types of high risk-human papillomavirus (HR-HPV) (16, 18, 31, 33, 35, 39, 45, 51, 52, 56, 58, 59, and 68) using Hybrid Capture II.

^b^ One-year and 2-year HR-HPV persistence were defined as HPV positivity in the 1-year follow-up study year and as HPV positivity in both the 1- and 2-year follow-up study years, respectively, after enrollment with HR-HPV positivity. One- and 2-year HPV negatives were defined as HPV negativity in the 1-year follow-up study year and as HPV negativity in both the 1- and 2-year follow-up study years, respectively, after enrollment with HPV negativity.

^c^ Non-SHS indicates persons who are not exposed to secondhand smoking at home or in the workplace, regardless of active smoking status.

^d^ Secondhand smoking (SHS) in nonsmokers was defined as having never smoked in life but having been exposed to SHS.

^e^ All variables were adjusted for age, BMI, marital status, oral contraceptive use, menopausal status, education level, and number of children.

^f^ The *p* value represents the multiplicative interaction of two factors.

## Data Availability

The datasets analyzed in this study are available from the corresponding author (Mi Kyung Kim, alrud@ncc.re.kr) upon reasonable request.

## Supplementary Materials

Supplementary Materials Supplementary Table 1: general characteristics of study subjects. Numbers are based on available data, as not all 9,846 women completely filled in the questionnaire, having excluded some variables related to smoking status. ^a^ Enrolment HR-HPV infection indicates the HR-HPV infection at the time of enrollment. “Negative” or “Positive” indicates the result of detection for 13 DNA types of high risk-human papillomavirus (HR-HPV) (16, 18, 31, 33, 35, 39, 45, 51, 52, 56, 58, 59, and 68) using Hybrid Capture II. ^b^ One-year and 2-year HR-HPV persistence were defined as HPV positivity in the 1-year follow-up study year and as HPV positivity in both the 1- and 2-year follow-up study years, respectively, after enrollment with HR-HPV positivity. One- and 2-year HPV negatives were defined as HPV negativity in the 1-year follow-up study year and as HPV negativity in both the 1- and 2-year follow-up study years, respectively, after enrollment with HPV negativity. ^c^ The chi-square test and* t*-test were used to assess the significance of differences in the distribution of categorical variables and continuous variables between the two groups, respectively. Supplementary questionnaire: standardized questionnaire with information on smoking and secondhand smoking.
