# Supplementary material for: Combined Effect of Secondhand Smoking and Alcohol Drinking on Risk of Persistent Human Papillomavirus Infection
Source: Biomed Res Int. 2019 Mar 21;2019:5829676. doi: 10.1155/2019/5829676 (PMC6448343; doi:10.1155/2019/5829676)
Supplement: Supplementary Materials — Supplementary Table 1: general characteristics of study subjects. Numbers are based on available data, as not all 9,846 women completely filled in the questionnaire, having excluded some variables related to smoking status. a Enrolment HR-HPV infection indicates the HR-HPV infection at the time of enrollment. “Negative” or “Positive” indicates the result of detection for 13 DNA types of high risk-human papillomavirus (HR-HPV) (16, 18, 31, 33, 35, 39, 45, 51, 52, 56, 58, 59, and 68) using Hybrid Capture II. b One-year and 2-year HR-HPV persistence were defined as HPV positivity in the 1-year follow-up study year and as HPV positivity in both the 1- and 2-year follow-up study years, respectively, after enrollment with HR-HPV positivity. One- and 2-year HPV negatives were defined as HPV negativity in the 1-year follow-up study year and as HPV negativity in both the 1- and 2-year follow-up study years, respectively, after enrollment with HPV negativity. c The chi-square test and t-test were used to assess the significance of differences in the distribution of categorical variables and continuous variables between the two groups, respectively. Supplementary questionnaire: standardized questionnaire with information on smoking and secondhand smoking. [file 5829676.f1.zip › 5829676_SupplDesc.docx.docx]

Supplementary table 1. General characteristics of study subjects

Numbers are based on available data, as not all 9,846 women completely filled-in the questionnaire, having excluded some variables related to smoking status.

a Enrolment HR-HPV infection indicates the HR-HPV infection at the time of enrolment.

‘Negative' or 'Positive' indicates the result of detection for 13 DNA types of high risk-human papillomavirus (HR-HPV) (16, 18, 31, 33, 35, 39, 45, 51, 52, 56, 58, 59, and 68) using hybrid capture II.

b 1-year and 2-year HR-HPV persistence were deﬁned as HPV positivity in the 1-year follow-

up study year and as HPV positivity in both the 1- and 2-year follow-up study years, respectively, after enrolment with HR-HPV positivity. One- and 2-year HPV negatives were deﬁned as HPV negativity in the 1-year follow-up study year and as HPV negativity in both the 1-and 2-year follow-up study years, respectively, after enrolment with HPV negativity.

c The Chi-square test and t-test were used to assess the significance of differences in the

distribution of categorical variables and continuous variables between the two groups, respectively.

Supplementary questionnaire: Standardized questionnaire with information on smoking and secondhand smoking

1
