# Supplementary material for: Combined Effect of Secondhand Smoking and Alcohol Drinking on Risk of Persistent Human Papillomavirus Infection
Source: Biomed Res Int. 2019 Mar 21;2019:5829676. doi: 10.1155/2019/5829676 (PMC6448343; doi:10.1155/2019/5829676)
Supplement: Supplementary Materials — Supplementary Table 1: general characteristics of study subjects. Numbers are based on available data, as not all 9,846 women completely filled in the questionnaire, having excluded some variables related to smoking status. a Enrolment HR-HPV infection indicates the HR-HPV infection at the time of enrollment. “Negative” or “Positive” indicates the result of detection for 13 DNA types of high risk-human papillomavirus (HR-HPV) (16, 18, 31, 33, 35, 39, 45, 51, 52, 56, 58, 59, and 68) using Hybrid Capture II. b One-year and 2-year HR-HPV persistence were defined as HPV positivity in the 1-year follow-up study year and as HPV positivity in both the 1- and 2-year follow-up study years, respectively, after enrollment with HR-HPV positivity. One- and 2-year HPV negatives were defined as HPV negativity in the 1-year follow-up study year and as HPV negativity in both the 1- and 2-year follow-up study years, respectively, after enrollment with HPV negativity. c The chi-square test and t-test were used to assess the significance of differences in the distribution of categorical variables and continuous variables between the two groups, respectively. Supplementary questionnaire: standardized questionnaire with information on smoking and secondhand smoking. [file 5829676.f1.zip › questionnaire (Eng. ver) (1).pdf]

Demographic Questionnaire

ID		Visit date	
Name		Date of birth	
Gender	Male · Female	Age	
Phone number		Telephone number	( ) -
Height	______cm	Weight	______kg
Address	(zip code: -)		

국립암센터
NATIONAL CANCER CENTER

1. What is your current marital status?

① Never married ☐ ② Married or living as married ☐ ③ Divorced ☐
④ Separated ☐ ⑤ Widowed ☐

2. What is your religion?

① Protestant ☐ ② Catholic ☐ ③ Buddhism ☐ ④ Hinduism ☐ ⑤ Islam ☐
⑥ Other ☐ ⑦ No religion ☐

3. Which of these categories best describe your current working situation ? If you are currently retired, please select about your previous retirement.

① Civil servant, soldier ☐
② Businessmen, management ☐
③ Office and technical staff ☐
④ Sales and service ☐
⑤ Production, labor ☐
⑥ Profession (professor, doctor, etc.) ☐
⑦ Self-employed ☐
⑧ Student ☐
⑨ Housewives (housework) ☐
⑩ Unemployed ☐
⑪ Not applicable (under 14 years old) ☐
⑫ Do not know ☐

4. What is your current job status?

① Regular wage worker ☐ ② Non-regular workers wage worker ☐
③ Employer with employee ☐ ④ Self-employed without employees ☐
⑤ Unpaid family worker ☐ ⑥ Not applicable ☐ ⑦ Do not know ☐

5. What is the highest grade or level of schooling you completed?

① Preschool children ☐ ② No school (unable to read Korean) ☐
③ No school (can read Korean) ☐ ④ less than 6 years ☐
⑤ 7 through 9 years ☐ ⑥ 10 through 12 years ☐
⑦ College graduate ☐ ⑧ Postgraduate ☐ ⑨ Do not know ☐

6. What is the average monthly household income (total income of all living together, including yourself)?

① Less than 1 million won ☐ ② Less than 2 million won ☐ ③ Less than 3 million won ☐
④ Less than 4 million won ☐ ⑤ Less than 5 million won ☐ ⑥ Less than 6 million won ☐
⑦ Less than 7 million won ☐ ⑧ Less than 8 million won ☐ ⑨ More than 8 million won ☐
⑩ Do not know ☐

※ Please answer the following questions only for women. (man is to be moved to question 14)

7. How old were you when you had your first menstrual period?

① _______ years ② Do not know ☐ ③ No menstruation yet ☐ (reason:)

8. Has you menstruation been completely suspended at this time?

① No ☐
1) Is the menstrual cycle regular?
① regular (every_____days) ② Irregular
2) Is the menstruation term normal?
① regular (every_____days) ② Irregular
3) When was the last menstrual start date? ___ month ____date

② Yes ☐
1) At what age did your the menstruation completely stopped?
______ year
2) Why was the menstruation stopped?
① Natural menopause ☐ ② Surgery ☐
③ Radiation ☐ ④ Drug therapy ☐
⑤ Do not know ☐
3) Have you ever taken or been injected with a female hormone since menopause ?
① Yes ☐ ② No ☐

9. Have you ever given birth?

① No ☐
② Yes ☐ 1) When was the first child born? ________year
2) How many children you have given birth ________person

10. Have you ever breastfeed your baby?

① No ☐
② Yes ☐ 1) ________person

11. Have your ever had a miscarriage?

① No ☐
② Natural heritage ☐ (_____times)
③ Artificial abortion ☐ (_____times)

12. Have you ever taken a contraceptive pill?

① No, never taken ☐

② Yes, still taking ☐ for __ year and ___ months from the age of ___

③ Yes, taken in the past ☐ for __ year and ___ months from the age of ___

13. Have you ever had hormone injections or medication since your menstruation has completely stopped (including both artificial and natural interruptions) ?

① No, never treated ☐

② Yes, still being treated ☐ for __ year and ___ months from the age of ___

③ Yes, treated in the past ☐ for __ year and ___ months from the age of ___

※ The following questions will be asked about your medical history and drug use.

14. Have you ever been diagnosed with the following disease from your doctor or clinic? Please refer to the next pages.

① No ☐

② Yes ☐ ☞ Please mark the item below.

Disease name	Age at the first diagnosis	Treatment status
☐ Hypertension	( )year	☐Complete recovery ☐Under treatment ☐Not treated
☐ Diabetes	( )year	☐Complete recovery ☐Under treatment ☐Not treated
☐ Thyroid disease	( )year	☐Complete recovery ☐Under treatment ☐Not treated
☐ Endometritis	( )year	☐Complete recovery ☐Under treatment ☐Not treated
☐ Breast positive disease	( )year	☐Complete recovery ☐Under treatment ☐Not treated
☐ Malignant tumor (cancer) Type:( )	( )year	☐Complete recovery ☐Under treatment ☐Not treated
☐ Malignant tumor (cancer) Type:( )	( )year	☐Complete recovery ☐Under treatment ☐Not treated
☐ Other chronic disease: ____________	( )year	☐Complete recovery ☐Under treatment ☐Not treated

Cancer type
1. Lip and oral 2. Esophageal 3. Gastric 4. Small bowel 5. Colorectal<br>6. Liver 7. Gallbladder 8. Biliary 9. Pancreatic 10. Laryngeal 11. Lung<br>12. Bone, articular cartilage 13. Skin 14. Mesothelial tumor tissue<br>15. Breast 16. Cervical 17. Endometrial 18. Overian 19. Prostate 20. Kidney<br>21. Renal pelvic 22. Ureteral 23. Bladder 24. Brain 25. Non-Hodgkin' s lymphoma<br>26. Multiple myeloma 27. Leukemia 28. Thyroid 29. Testicular 30. Other

15. Have your immediate family member (parents, siblings, children) or relatives (blood relatives) been diagnosed with the following disease from a doctor or clinic ?

① No ☐

② Yes ☐ ☞ Please mark the item below.

Disease name	If yes, please indicate all cases
☐ Hypertension	☐Grandfather (father' s side) ☐Grandmother (father' s side)<br>☐Grandfather (mother' s side) ☐Grandmother (mother' s side)<br>☐Father ☐Mother ☐Brothers ☐Relative:____☐Relative: ___
☐ Diabetes	☐Grandfather (father' s side) ☐Grandmother (father' s side)<br>☐Grandfather (mother' s side) ☐Grandmother (mother' s side)<br>☐Father ☐Mother ☐Brothers ☐Relative:____☐Relative: ___
☐ Stroke	☐Grandfather (father' s side) ☐Grandmother (father' s side)<br>☐Grandfather (mother' s side) ☐Grandmother (mother' s side)<br>☐Father ☐Mother ☐Brothers ☐Relative:____☐Relative: ___
☐ Heart diease	☐Grandfather (father' s side) ☐Grandmother (father' s side)<br>☐Grandfather (mother' s side) ☐Grandmother (mother' s side)<br>☐Father ☐Mother ☐Brothers ☐Relative:____☐Relative: ___
☐ Cervical cancer	☐Grandfather (father' s side) ☐Grandmother (father' s side)<br>☐Grandfather (mother' s side) ☐Grandmother (mother' s side)<br>☐Father ☐Mother ☐Brothers ☐Relative:____☐Relative: ___
☐ Thyroid cancer	☐Grandfather (father' s side) ☐Grandmother (father' s side)<br>☐Grandfather (mother' s side) ☐Grandmother (mother' s side)<br>☐Father ☐Mother ☐Brothers ☐Relative:____☐Relative: ___
☐ Breast cancer	☐Grandfather (father' s side) ☐Grandmother (father' s side)<br>☐Grandfather (mother' s side) ☐Grandmother (mother' s side)<br>☐Father ☐Mother ☐Brothers ☐Relative:____☐Relative: ___
☐ Other cancer:<br>______________	☐Grandfather (father' s side) ☐Grandmother (father' s side)<br>☐Grandfather (mother' s side) ☐Grandmother (mother' s side)<br>☐Father ☐Mother ☐Brothers ☐Relative:____☐Relative: ___
☐ Other chronic diease:<br>______________	☐Grandfather (father' s side) ☐Grandmother (father' s side)<br>☐Grandfather (mother' s side) ☐Grandmother (mother' s side)<br>☐Father ☐Mother ☐Brothers ☐Relative:____☐Relative: ___

16. Do you have medicines that have been taken twice or more per week over the last two years?

① There is no medicine to eat constantly ☐
② Yes ☐ ☞ Please mark below.

Drug name	Number of times	Single dose	Total duration
☐ Acetaminophen	weekly or daily ___ times	____pills	_____month
☐ Aspirin	weekly or daily ___ times	____pills	_____month
☐ Blood pressure medicine	weekly or daily ___ times	____pills	_____month
☐ Diabetes drug	weekly or daily ___ times	____pills	_____month
☐ Allergy drug	weekly or daily ___ times	____pills	_____month
☐ Heartburn medication	weekly or daily ___ times	____pills	_____month
☐ Other drugs name: _______________	weekly or daily ___ times	____pills	_____month
☐ Other drugs name: _______________	weekly or daily ___ times	____pills	_____month

※ The following section is for smoking and drinking habits.

17. Have you ever smoked cigarettes ?

① No ☐
② Yes, still smoking ☐

1) How old were you when you first smoked? _______year
2) How many years have you smoked so far? _______year
3) How many cigarettes have you smoked per day on average in the past year? ________cigarettes

③ Yes, but do not smoke now. ☐
1) If you quit smoking, how long is it?
☐Less than 1 year ☐1-3 years ☐4-6 years ☐7-10 years
☐11-15 years ☐More than 16 years ☐ Do not know
2) Before you quit smoking, how many cigarettes did you smoke on average a day in year? _______cigarettes

18. Have you ever been exposed to smoking indirectly?

① No ☐
② Yes ☐

1) How much are you exposed to smoking at home?

☐ None ☐ Less than 3 days per week
☐ More than 3 days per week ☐ Daily

2) How long are you exposed to average daily smoking in your home ?

________hours ________minutes

3) How long are you exposed to smoking at work?

☐ None ☐ Less than 3 days per week
☐ More than 3 days per week ☐ Daily

4) How long are you exposed to average daily smoking in your work?

________hours ________minutes

19. Have you ever had any drinking experience?

① No ☐

② Yes ☐

1) How many years have you been drinking so far? ________years

2) Please indicate the average number of drinks during the past year.

☐less than once a month ☐1-3 times a month ☐ 1-2 times a week
☐ 3-4 times a week ☐ 5-6 times a week ☐ daily

③ No, but do not drink now ☐

1) How long have you stopped drinking ? ________years

2) Please indicate the average number of times you drunk during a year before you stop drinking.

☐less than once a month ☐1-3 times a month ☐ 1-2 times a week
☐ 3-4 times a week ☐ 5-6 times a week ☐ daily

※ The following survey is to evaluate your regular exercise habits during the past 7 days.

20. How many days did you do intense physical activity during the last 7 days?

① ________ days / week
② No intense physical activity ☞*Go to question22*

21. How often did you usually do intense physical activity during the day?

① ______ hours _________minutes
② Do not know

22. How many days did you perform moderate physical activity during the last 7 days?

① ________ days / week
② No moderate physical activity ☞*Go to question24*

23. How often did you have a moderate amount of physical activity during the day?

① ______ hours _________minutes
② Do not know

24. How many days did you walk for a least 10 minutes at a time during the last 7 days?

① ________ days / week
② Never walked ☞*Go to question26*

25. How often did you walk during the day?
① ______ hours _________minutes
② Do not know

26. How much time have you spent sitting on weekdays during the last 7 days?
① ______ hours _________minutes
② Do not know
