# Supplementary material for: Combined Effect of Secondhand Smoking and Alcohol Drinking on Risk of Persistent Human Papillomavirus Infection
Source: Biomed Res Int. 2019 Mar 21;2019:5829676. doi: 10.1155/2019/5829676 (PMC6448343; doi:10.1155/2019/5829676)
Supplement: Supplementary Materials — Supplementary Table 1: general characteristics of study subjects. Numbers are based on available data, as not all 9,846 women completely filled in the questionnaire, having excluded some variables related to smoking status. a Enrolment HR-HPV infection indicates the HR-HPV infection at the time of enrollment. “Negative” or “Positive” indicates the result of detection for 13 DNA types of high risk-human papillomavirus (HR-HPV) (16, 18, 31, 33, 35, 39, 45, 51, 52, 56, 58, 59, and 68) using Hybrid Capture II. b One-year and 2-year HR-HPV persistence were defined as HPV positivity in the 1-year follow-up study year and as HPV positivity in both the 1- and 2-year follow-up study years, respectively, after enrollment with HR-HPV positivity. One- and 2-year HPV negatives were defined as HPV negativity in the 1-year follow-up study year and as HPV negativity in both the 1- and 2-year follow-up study years, respectively, after enrollment with HPV negativity. c The chi-square test and t-test were used to assess the significance of differences in the distribution of categorical variables and continuous variables between the two groups, respectively. Supplementary questionnaire: standardized questionnaire with information on smoking and secondhand smoking. [file 5829676.f1.zip › [F] supplement table1.pdf]

Supplementary table 1. General characteristics of study subjects

Characteristics	HR-HPV infection at enrollment		p value [c]	1-year HR-HPV persistence[b]		p value [c]	2-year HR-HPV persistence[b]		p value [c]
	Negative (N = 8,866)	Positive (N = 980)		Negative (N = 1,092)	Persistence (N = 145)		Negative (N = 432)	Persistence (N = 49)	
Age									
Mean (SE)	48.2 (0.10)	46.9 (0.29)	<0.01	48.4 (0.25)	46.8 (0.73)	0.04	48.0 (0.38)	48.1 (1.05)	0.91
Body mass index									
Mean (SE)	23.0 (0.03)	22.7 (0.10)	0.02	22.8 (0.09)	22.4 (0.23)	0.10	22.6 (0.14)	21.7 (0.31)	0.04
Marriage									
Single	239 (86.6)	37 (13.4)	0.06	15 (71.4)	6 (28.6)	0.02	5 (88.3)	1 (16.7)	0.56
Married	8145 (90.1)	894 (9.9)		1011 (88.5)	131 (11.5)		399 (90.1)	44 (9.9)	
Menopausal									
No	2316 (88.6)	298 (11.4)	<0.01	235 (82.8)	49 (17.2)	0.02	89 (87.3)	13 (12.7)	0.56
Yes	3558 (91.2)	341 (8.8)		419 (88.6)	54 (11.4)		146 (89.6)	17 (10.4)	
No. of children									
None or one	936 (90.6)	97 (9.4)	0.86	116 (90.6)	12 (9.4)	0.61	45 (91.8)	4 (8.2)	0.72
Two	4229 (90.4)	448 (9.6)		523 (87.6)	74 (12.4)		204 (88.7)	26 (11.3)	
Three or more	2170 (90.1)	239 (9.9)		234 (88.6)	30 (11.4)		90 (90.9)	9 (9.1)	
Education level									
≤Middle school	1667 (91.3)	158 (8.7)	0.04	155 (86.6)	24 (13.4)	0.29	54 (93.1)	4 (6.9)	0.38
High school	3450 (90.2)	374 (9.8)		432 (87.1)	64 (12.9)		174 (87.9)	24 (12.1)	
≥University	3137 (89.2)	380 (10.8)		422 (90.0)	47 (10.0)		177 (91.2)	17 (8.8)	
Active smoke									
Non-smoker	8145 (90.3)	876 (9.7)	<0.01	1002 (88.5)	130 (11.5)	0.39	388 (89.4)	46 (10.6)	0.36
Smoker (Current/Past)	721 (87.4)	104 (12.6)		90 (85.7)	15 (14.3)		44 (93.6)	3 (6.4)	
Secondhand smoke									
Non-smoker	5628 (90.4)	601 (9.6)	0.19	672 (88.4)	88 (11.6)	0.84	251 (89.6)	29 (10.4)	0.88
Smoker (Current/Past)	3238 (89.5)	379 (10.5)		420 (88.1)	57 (11.9)		181 (90.1)	20 (9.9)	
Alcohol consumption									
Non-drinker	4242 (91.2)	410 (8.8)	<0.01	580 (90.8)	59 (9.2)	< 0.01	231 (91.7)	21 (8.3)	0.07
Drinker (Current/Past)	4059 (88.7)	519 (11.3)		447 (84.8)	80 (15.2)		177 (86.3)	28 (13.7)	
Oral contraceptive use									
Never	5231 (89.9)	589 (10.1)	0.13	573 (86.4)	90 (13.6)	0.23	213 (87.7)	30 (12.3)	0.28
User (Current/Past)	1357 (91.2)	131 (8.8)		174 (89.7)	20 (10.3)		61 (92.4)	5 (7.6)	
Cytological results									
Normal	8189 (91.1)	800 (8.9)	<0.01	1055 (90.2)	115 (9.8)	< 0.01	423 (92.8)	33 (7.2)	<0.01
ASCUS	113 (60.8)	73 (39.2)		24 (60.0)	16 (40.0)		9 (50.0)	9 (50.0)	
LSIL	12 (20.7)	46 (79.3)		3 (21.4)	11 (78.6)		0 (0.0)	6 (100)	
HSIL or worse	2 (6.9)	27 (93.1)		0 (0.0)	0 (0.0)		0 (0.0)	0 (0.0)	

Numbers are based on available data, as not all 9,846 women completely filled-in the questionnaire, having excluded some variables related to smoking

status.

[a] Enrolment HR-HPV infection indicates the HR-HPV infection at the time of enrolment. ‘Negative' or 'Positive' indicates the result of detection for 13 DNA types of high risk-human papillomavirus (HR-HPV) (16, 18, 31, 33, 35, 39, 45, 51, 52, 56, 58, 59, and 68) using hybrid capture II.

[b] 1-year and 2-year HR-HPV persistence were defined as HPV positivity in the 1-year follow-up study year and as HPV positivity in both the 1- and 2-year follow-up study years, respectively, after enrolment with HR-HPV positivity. One- and 2-year HPV negatives were defined as HPV negativity in the 1-year follow-up study year and as HPV negativity in both the 1-and 2-year follow-up study years, respectively, after enrolment with HPV negativity.

[c] The Chi-square test and t-test were used to assess the significance of differences in the distribution of categorical variables and continuous variables between the two groups, respectively.
